# Field durability of the same type of long-lasting insecticidal net varies between regions in Nigeria due to differences in household behaviour and living conditions

**DOI:** 10.1186/s12936-015-0640-4

**Published:** 2015-03-24

**Authors:** Albert Kilian, Hannah Koenker, Emmanuel Obi, Richmond A Selby, Megan Fotheringham, Matthew Lynch

**Affiliations:** Tropical Health LLP, Montagut, Spain; Malaria Consortium, London, UK; Johns Hopkins Bloomberg School of Public Health Center for Communication Programs, Baltimore, MD USA; Malaria Consortium Nigeria Office, Abuja, Nigeria; Malaria Consortium Africa Office, Kampala, Uganda; United States Agency for International Development, President’s Malaria Initiative, Washington, DC USA

**Keywords:** Malaria prevention, ITN, Net durability, Nigeria

## Abstract

**Background:**

With the recent publication of WHO-recommended methods to estimate net survival, comparative analyses from different areas have now become possible. With this in mind, a study was undertaken in Nigeria to compare the performance of a specific long-lasting insecticidal net (LLIN) product in three socio-ecologically different areas. In addition, the objective was to assess the feasibility of a retrospective study design for durability.

**Methods:**

In three states, Zamfara in the north, Nasarawa in the centre and Cross River in the south, four local government areas were selected one year after mass distribution of 100-denier polyester LLINs. From a representative sample of 300 households per site that had received campaign nets, an assessment of net survival was made based on rate of loss of nets and the physical condition of surviving nets measured by the proportionate hole index (pHI). Surveys were repeated after two and three years.

**Results:**

Over the three-year period 98% of the targeted sample size of 3,720 households was obtained and 94% of the 5,669 campaign nets found were assessed for damage. With increasing time since distribution, recall of having received campaign nets dropped by 11-22% and only 31-87% of nets actually lost were reported. Using a recall bias adjustment, attrition rates were fairly similar in all three sites. The proportion of surviving nets in serviceable condition differed dramatically, however, resulting in an estimated median net survival of 3.0 years in Nasarawa, 4.5 years in Cross River and 4.7 years in Zamfara. Although repairs on damaged nets increased from around 10% at baseline to 21-38% after three years, the average pHI value for each of the four hole size categories did not differ between repaired and unrepaired nets.

**Conclusions:**

First, the differences observed in net survival are driven by living conditions and household behaviours and not the LLIN material. Second, recall bias in a retrospective durability study can be significant and while adjustments can be made, enough uncertainty remains that prospective studies on durability are preferable wherever possible. Third, repair does not seem to measurably improve net condition and focus should, therefore, be on improving preventive behaviour.

**Electronic supplementary material:**

The online version of this article (doi:10.1186/s12936-015-0640-4) contains supplementary material, which is available to authorized users.

## Background

While further progress is made towards achieving universal coverage with insecticide-treated nets (ITNs) in Africa south of the Sahara [1], increasing focus is given to the question of how to sustain these successes, particularly through improvements in field durability in long-lasting insecticidal nets (LLINs). This is not only important to determine the optimal time for net replacement in sustaining universal coverage [2], but also to obtain optimal cost-effectiveness in LLIN procurement (cost per year of use) [3].

The World Health Organization (WHO) has, in recent years, developed clear definitions and methodologies to assess net durability and survival in field conditions [4-6]. The guidance has led to a significant increase of studies presenting comprehensive and comparable durability assessments in different regions [7-9] and for different products [10,11], which suggest considerable variation in net survival ranging from less than two years to four or more years. In addition, quantitative [9,12,13] as well as qualitative [14-16] data are becoming available to better understand the determinants of field performance with respect to environmental conditions and user behaviour, which demonstrate that such factors have at least as strong an influence as the physical specifications of the nets [17].

While the standard design for durability assessment is a prospective study where nets are identified at the time of distribution and then followed up over a given time or until they are lost [4], this is not always possible due to time or logistical constraints. Therefore, WHO guidelines also envisage a retrospective design where assessments are conducted using multiple cross-sectional surveys highlighting the potential of recall bias in this type of assessment [4]. The present study was undertaken in Nigeria to describe how field durability of a very similar LLIN product (100-denier polyester net) compares between three very different ecological and sociocultural areas of the country and to define the main determinants of potential differences. In addition, the study intended to explore the feasibility of such a retrospective approach to durability assessment, the level of bias involved and how this could be accounted for in the analysis.

## Methods

### Study design

This study was a multi-site, retrospective assessment of net durability and survival with three rounds of annual, representative cluster-sampling household surveys. At each survey round a representative sample of households that had received nets from the preceding campaign were included and the attrition (nets received and lost) and physical integrity of surviving nets measured. While clusters (settlements) remained the same over time, households were re-sampled at each round so that annual samples can be considered independent. Nested within this observational study was an intervention study on the impact of behaviour change communication (BCC) on net care and repair behaviour, the design and results of which are reported separately [18].

### Study sites

**Figure 1:**
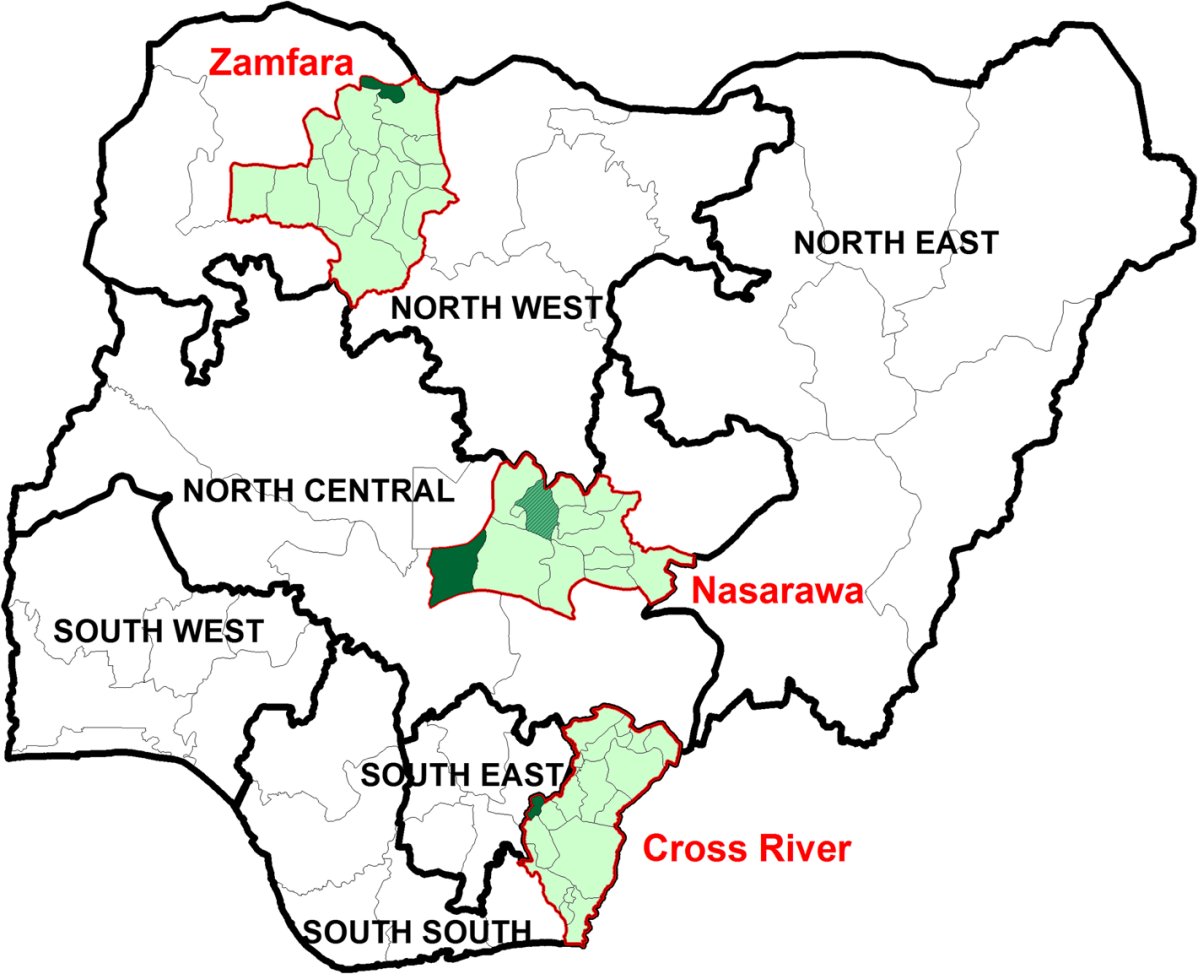
**Map of Nigeria showing the three study states and four local government areas (LGAs).** In Nasarawa solid shape = control site (Toto LGA), striped = intervention site (Kokona LGA).

Three states were purposively selected to represent three distinct ecological and climatic settings in Nigeria and in each one a rural local government area (LGA, equivalent to a district) was chosen as study site (Figure 1). Zamfara State is located in the dry savannah in the north (North West Zone) with an average annual rainfall of 600 mm between May and October (six months). The selected LGA was Shinkafe with an estimated 2012 population of 163,868 inhabitants. Nasarawa State is situated in the Guinea Savannah of central Nigeria (North Central Zone) with an average annual rainfall of 1,400 mm between March and November (nine months). The selected LGA was Toto (2012 population 142,184) with an additional LGA selected as the intervention site for the previously mentioned care and repair study (Kokona LGA, population 131,046). The third location was Cross River State in the southeast of the country (South East Zone) dominated by tropical primary and secondary forests of the Niger Delta and an average annual rainfall of 2,400 mm almost all year round (11 months). The selected site was Abi LGA with a 2012 population estimate of 171,896.

The three sites also differed in their sociocultural context: Zamfara has a majority Hausa ethnic group who are predominantly Muslim, while Cross River’s population is predominantly Efik ethnicity and Christian. Between these two states Nasarawa represents a mix of ethnic and cultural influences.

### Campaigns and campaign nets

Nigeria’s National Malaria Elimination Programme started the first round of mass campaigns for the distribution of LLINs to all households in 2009 in Kano State [19] and targeted two LLINs for every household registered by the mobilization teams. The three selected states had their campaign within three months of each other, starting with Nasarawa in December 2010, Cross River in January 2011 and Zamfara in March 2011. At all three sites the same or very similar LLIN product was used, a polyester LLIN with a 100-denier yarn strength which was Permanet 2.0® (Vestergaard) in Zamfara and Nasarawa, and DAWA Plus 2.0® (Tana Netting) in Cross River.

### Sampling and sample size

Sampling for the cross-sectional household surveys was done in two major steps. First, clusters defined as settlements (villages) were selected with probability proportionate to population size. Because no sampling frame was available for the village population, the population lists of wards (administrative unit below LGA) were used to allocate clusters and then within each selected ward an up-to-date list of all settlements was obtained from the state authorities and one settlement selected using simple random sampling. These clusters were maintained throughout the three annual survey rounds.

For the selection of households within each cluster and survey all households were mapped on the survey day and the required number selected using random number lists. If a community had more than 200 households it was divided into approximately equal sections with the help of local leaders and one of the sections randomly selected for mapping. A household was defined as all people ‘eating from the same pot’. Each selected household was then screened to assess whether they had participated in the mass campaign and received any nets. If they had not received any campaign nets, the household was dropped and a replacement household from the random list selected. Up to ten replacements were available per cluster.

For the calculation of sample size, the following assumptions were made: alpha error of 0.05 (95% confidence intervals), beta error of 0.2 (power 80%), design effect of 1.75, non-response of 5% and an average of 1.8 nets received per household that participated in the campaign. Based on the calculations a sample of 20 clusters with 15 households (300 households per site and time point) was determined as sufficient, resulting in a sample of 597 campaign nets to be assessed per site in year one, 498 in year two and 332 in year three. Assuming a net survival of 50% after three years, the expected precision of the survival estimate was ±5.6 percentage-points, sufficient to detect a 11.2% difference in survival between the sites at the end of the study, which was considered programmatically relevant. For the last survey round (year three) the number of clusters in Toto LGA, Nasarawa, was increased to 28 (420 households) in order to increase power for the nested care and repair study due to some contamination of intervention (radio) into the control area [18].

### Field procedures

For each state, three teams of three interviewers and one supervisor were selected and trained, as well as one overall state coordinator. These teams largely stayed together throughout the three-year study period with very little fluctuation. Teams were trained for one week before each survey. The training consisted of two parts: first, training on the survey sampling and interviewing procedures with detailed work on the questionnaire and how it would be used in the local languages in a standardized fashion. This was followed by a theoretical and practical training on the assessment of the physical condition of nets following a training manual previously developed based on other durability studies and using a template for the determination of hole size categories and a tally sheet to assist in counting the number of holes on the net [20]. Campaign nets were identified using a visual aid of labels and packaging of all available LLIN brands.

A structured questionnaire was used to gather data on household characteristics, nets received from mass campaigns, any nets lost and the reasons for the loss, net care and repair behaviour and attitudes, exposure to care and repair messages, and characteristics as well as assessment of existing campaign nets. Holes in the nets were categorized into four distinct groups as recommended by WHO [4]: size one (0.5-2 cm in diameter), size two (2–10 cm), size three (10–25 cm) and size four (larger than 25 cm). The presence and number of repairs were also counted on each net. Respondents with nets that had any signs of damage were asked how any of the holes had occurred and five categories were recorded allowing multiple responses: “torn on an object”, “pulled and torn”, “seam came open” (summarized as mechanical damage), “damage from mice or rats” (rodent damage), and “burns from flame or sparks” (thermal damage). Some modifications of the questionnaire were made for the third survey round with more detailed questions being added with respect to attitudes and practices regarding net durability, care and repair.

All surveys were done between March and April in the three years following the campaign (2012–2014) with the exact time elapsed since the campaign varying between 1.1 and 1.2 years for the first round, 2.1 to 2.3 years for the second round and 3.1 to 3.3 years at the third round (see Additional file 1).

### Data preparation and analysis

Data were collected on paper forms in the field and then entered by qualified staff into an EpiData 3.1 data base (EpiData Association, Odense, Denmark) using double entry and record validation. The cleaned versions of the datasets were then transferred for further processing and analysis to the Stata 13.1 software package (Stata Corp., College Station, TX, USA).

Assessment of the physical integrity of nets followed the most recent recommendations of WHO [4-6] and was based on a two-step approach. First, the proportionate hole index (pHI) was calculated for each net based on the number of holes in each size category and multiplying them with the recommended weights:

$$ \mathrm{pHI} = \left(\#\mathrm{size}\ 1\ \mathrm{holes}\right) + \left(\#\mathrm{size}\ 2\ \mathrm{holes} \times 23\right) + \left(\#\mathrm{size}\ 3\ \mathrm{holes} \times 196\right) + \left(\#\mathrm{size}\ 4\ \mathrm{holes} \times 578\right). $$

Based on the pHI value nets were then divided into three categories:

- Good: total hole surface area <0.01 sq m or pHI <64
- Acceptable: total hole surface area >0.01 < =0.1 sq m or pHI >64 < =642
- Torn: total hole surface area >0.1 sq m or pHI >642

The first two categories were then combined as:

- Serviceable: Net is either good or acceptable

The rate of attrition was calculated as the proportion of campaign nets lost among all nets originally received. Based on the reported reasons for net loss, attrition was further divided into: i) due to “wear and tear” defined as discarding of nets by throwing away, destroying or using them for other purposes, since previous data from a multi-country analysis has shown that these re-purposed nets are predominantly torn and considered no longer usable [21]; and, ii) due to giving them away for others to use [4]. The functional survival to time point x was calculated as the number of campaign nets still in serviceable condition at time x divided by the number originally received and not given away (i.e., surviving nets plus loss to “wear and tear”) [5].

Based on the considerable recall bias observed in the data (Table 1) an adjustment was made for the estimation of campaign nets received and lost. Details of the calculations are shown in Additional file 2. In short, the number of campaign nets reported as received after two and three years, respectively, was inflated by the ratio between reported nets received per person in the household in that survey compared to the first one (one year after the campaign). The number of campaign nets lost was taken as the difference between campaign nets received and those actually seen in the survey and identified as campaign nets by LLIN brand. The number lost to “wear and tear” was obtained by applying the proportion from the data to the adjusted number of nets lost. This adjustment for lost nets was done for all three survey rounds.

**Table 1:** **Magnitude of recall bias for campaign nets received and lost**

**Location**	**Recall of campaign nets received**				**Recall of campaign nets lost**	
**Year**	**Mean persons per HH**	**Mean nets reported received**	**Nets per person reported**	**% of first year**	**Actual lost (received – present)**	**Reported lost (% of actual)**
Zamfara						
Year 1	8.33	2.35	0.2821	100%	47	41 (87.2%)
Year 2	7.76	1.86	0.2397	84.9%	70	37 (52.9%)
Year 3	7.99	1.77	0.2215	78.5%	84	26 (31.0%)
Nasarawa						
Year 1	8.39	1.73	0.2062	100%	243	144 (59.3%)
Year 2	8.71	1.71	0.1963	95.2%	162	83 (51.2%)
Year 3	8.52	1.57	0.1843	89.4%	293	211 (72.0%)
Cross River						
Year 1	5.36	1.63	0.3046	100%	75	40 (53.3%)
Year 2	5.71	1.40	0.2452	80.5%	69	35 (50.7%)
Year 3	5.55	1.41	0.2541	83.4%	70	25 (35.7%)

HH **=** household.

Nets received are measured by the nets reported received per person in the household compared to the first year results while nets lost are evaluated by comparing the reported number with the difference between nets received and actually observed.

Following the recommendations of WHO [5], the estimated net survival was plotted against hypothetical survival curves with defined median survival times [6] and details of these functions are found in Additional file 3.

Median estimated net survival was calculated from at least two time points, the lowest of which was below 85% using the following formula:

$$ tm=t1+\frac{\left(t2-t1\right)\times \left(p1-50\right)}{\left(p1-p2\right)} $$

where tm is the median survival time, t1 and t2 the first and second time points in years and p1 and p2 the proportion surviving to first and second time point respectively in per cent. The confidence interval of the median net survival was obtained by applying the formula to the lower and upper limits of p1 and p2, respectively.

To capture care and repair attitudes of households, an attitude score was constructed based on responses to eight statements introduced at the third survey round. These statements used a four-level Likert scale, where 1 was “strongly disagree” and 4 was “strongly agree”. These were recoded during analysis to have −2 be “strongly disagree” and +2 be “strongly agree”. Two statements were negatively phrased, and therefore were inversely recoded to make a positive response +2. Attitude scores for each respondent were summed and divided by eight to calculate an overall attitude score. Scores were then categorized into four groups: equal or less than zero (negative attitude); 0.01-0.74 (somewhat positive attitude); 0.75-1.49 (positive attitude); and, 1.50-2.00 (very positive attitude).

The wealth index was computed at the household level using principal component analysis (PCA). The variables for household amenities, assets, livestock, and other characteristics that are related to a household’s socio-economic status were used for the computation. All variables were dichotomized except those of animal ownership where the total number owned was used. The first component of the PCA was used as the wealth index. Households were then classified according to their index value into quintiles within each study site and time point.

For all statistical analyses the cluster design was taken into account by applying the survey family of commands and thereby adjusting confidence intervals (CI) for the design effect. The CI for the adjusted net survival rates were obtained by calculating the exact binomial 95% CI from the adjusted numerator and denominator and then inflating the CI by the design effect obtained from the data for the proportion of campaign nets in serviceable conditions.

In order to account for potential confounders in the analysis of key outcomes multivariate regression models were used, logistic regression for dichotomous outcomes and linear regression for continuous variables. Models were constructed using backwards elimination and Wald tests for significant parameters. Variables that define the data structure such as site and time point were included in all models irrelevant of significance level.

Because the data from the nested care and repair study did not show a significant difference between the control site (Toto LGA) and the intervention site (Kokona LGA) due to the radio contamination [18] both sites were included for Nasarawa in the durability analysis.

### Ethical clearance

Ethical approval was obtained from the Johns Hopkins School of Public Health Institutional Review Board (IRB #4108) and from the National Health Research Ethics Committee, Federal Ministry of Health in Nigeria (NHREC/01/01/2007). Respondents were informed about the purpose of the study in the dominant local language (Hausa or Efik) using a written script and the interview proceeded when verbal consent was given. This consent form contained information on the objectives of the survey, the risks, benefits and freedom of the participation, as well as information on confidentiality plus respondent rights.

## Results

### The sample

Out of a total of 3,720 households to be sampled according to protocol, 3,649 (98.1%) valid interviews from households that had received campaign nets were obtained with a range by year and site between 92.0 and 100% (see Additional file 1). The number of campaign nets found in the sampled houses was 5,669 in total but decreased over time. In the first survey, the average number of campaign nets per sampled household was 1.79, very close to the 1.8 assumed for the sample size calculations. This rate decreased to 1.53 nets per household in the second survey round and 1.37 in the third. Field teams were able to assess 93.8% of all campaign nets for physical damage and the range across sites and surveys was between 82.6 and 99.2% (see Additional file 1).

As anticipated by the study design, house characteristics, assets and sociodemographic variables differed significantly between sites with a north–south gradient for many of the variables (see Additional file 4). At the Zamfara site the vast majority of houses had thatch or grass roofs, mud walls and floors made from earth or clay. In contrast, at the Cross River site more than 70% of houses had roofs made from sheets (iron or aluminium), plastered or brick walls and tile floors. The Nasarawa site was mixed, with mostly sheet roofs but only 43% plastered walls and 42% of houses had earth floors. Ownership of mobile phones or television sets increased from the north to centre to south with the exception of radios, which was lower in Cross River as 65% of households owned a TV set. Education also showed a strong gradient with only 17% of heads of household at the Zamfara site having had at least some secondary school while this was 27% in Nasarawa and 45% in Cross River. The north had a higher proportion of polygamous households, more households with children under five and a higher child density (child to adult ratio). Mean household size was around eight persons in Zamfara and Nasarawa and between five and six in Cross River.

### Attrition, integrity and survival

Table 1 presents the level of recall bias for campaign nets received and lost documented in the surveys. Compared to the first survey, one year after the campaign, respondents’ recall of the number of nets received from the campaign at year three reduced by 21.5% in Zamfara, by 16.6% in Cross River and by 10.6% in Nasarawa. The recall bias for nets lost was even larger, with generally only half of the nets lost based on the difference between those received and found in the survey reported by the respondents. In Zamfara and Cross River the recall worsened from the first to the third survey while in Nasarawa, the site of the nested care and repair BCC impact study, recall was highest at the last survey with 72%.

Results from the attrition and net integrity analysis are summarized in Table 2. The loss of campaign nets for any reason over time was very similar at all three sites with attrition rates – adjusted for recall bias – increasing from between 6 and 22% in year one to between 29 and 34% in year three. Among the lost nets the proportion that were discarded due to “wear and tear” increased over time at all sites and was very similar in Nasarawa and Cross River increasing from 34% in year one to 55 and 64%, respectively, in year three. In comparison to these two states a larger proportion of lost nets was given away for others to use in Zamfara with only 5% of all lost nets reported to have been discarded in year one, 25% in year two and 40% in year three. Considering only the discarded or re-purposed nets, the attrition rate for campaign nets due to “wear and tear” after three years was 13% in Zamfara, 18% in Nasarawa and 19% in Cross River.

**Table 2:** **Attrition and integrity of campaign nets up to three years after distribution**

**Location**	**Attrition (adjusted)**		**Integrity (physical condition)**	
**Year**	All*	Due to “wear and tear”**	**Good (95% CI)**	**Serviceable (95% CI)**
Zamfara				
Year 1	6.0%	0.3%	94.7% (92.3, 96.4)	98.5% (96.9, 99.2)
Year 2	25.6%	6.5%	76.3% (69.4, 82.0)	93.9% (90.6, 96.0)
Year 3	33.6%	13.4%	60.5% (53.8, 66.7)	89.9% (86.4, 92.5)
Nasarawa				
Year 1	22.2%	7.5%	80.9% (75.5, 85.2)	92.3% (89.3, 94.5)
Year 2	19.0%	9.6%	51.6% (44.6, 58.5)	72.6% (66.8, 77.8)
Year 3	32.3%	18.0%	31.7% (25.5, 38.6)	53.1% (46.9, 59.3)
Cross River				
Year 1	13.5%	4.7%	93.7% (89.3, 96.4)	97.7% (95.5, 98.9)
Year 2	31.2%	12.5%	83.8% (76.3, 89.2)	93.1% (88.1, 96.1)
Year 3	28.9%	18.6%	74.5% (67.8, 80.2)	88.4% (83.8, 91.9)

*All nets lost since distribution irrespective of reason for loss, i.e. including nets given away for other so use.

**Nets received and discarded (destroyed, thrown away) or used for other purposes than sleeping under.

Attrition is reported as all cause and only due to discarding and re-purposing of nets; Integrity is based on the proportionate hole index (pHI) values (see Methods section for details).

The physical condition of surviving nets (Table 2) deteriorated significantly faster in Nasarawa with only slightly more than half of the nets still in serviceable condition after three years compared to 88% in Cross River and 90% in Zamfara.

**Figure 2:**
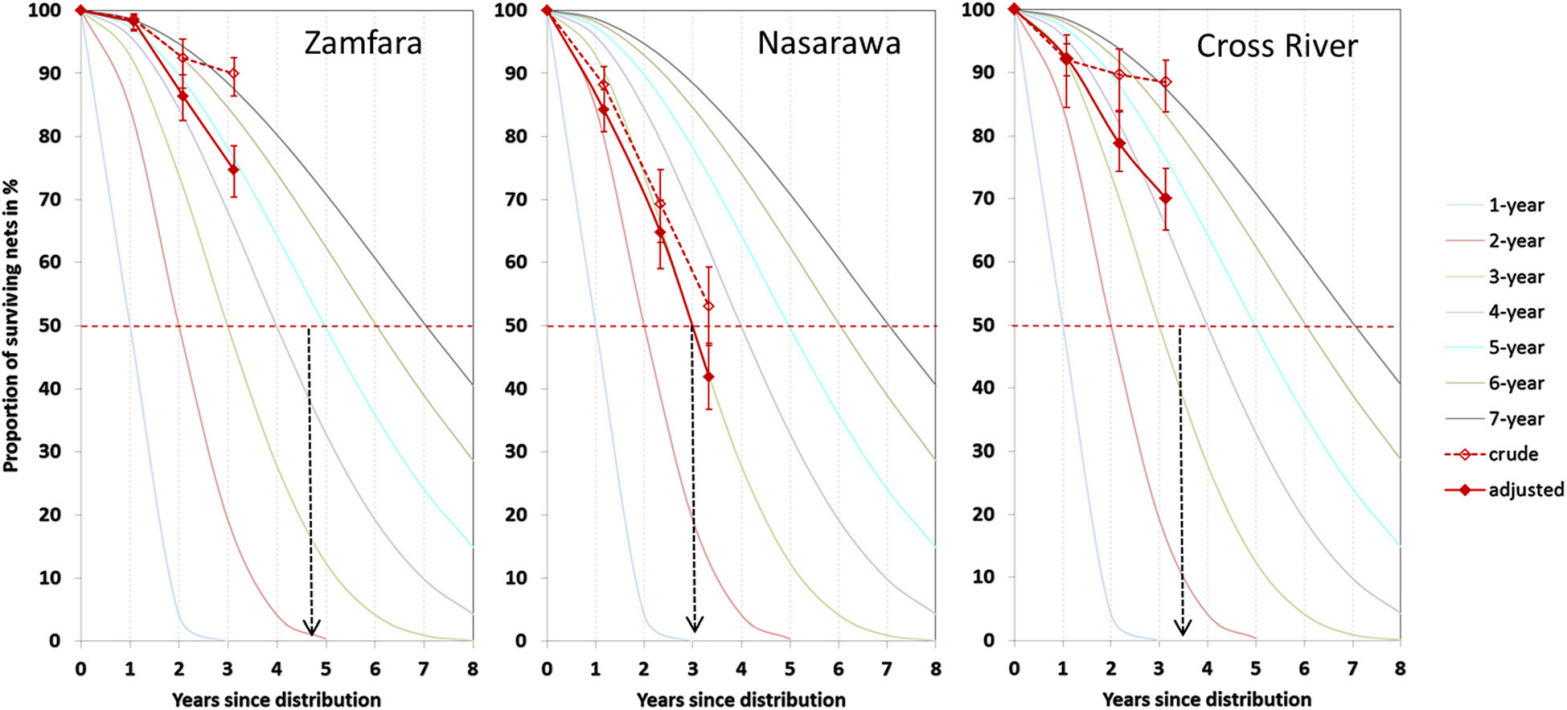
**Survival in functional condition of campaign nets (100-denier polyester LLIN) up to three years after distribution in comparison to hypothetical survival curves of defined median survival.** Solid lines = recall adjusted estimates; dashed line = crude estimates; horizontal dotted line = median survival; vertical arrows indicate where the functional survival curves reach or are projected to reach the median.

The resulting, estimated, functional survival of campaign nets is shown in Table 3 and Figure 2. There was a striking difference between the crude and recall-adjusted estimates of functional net survival especially in Zamfara and Cross River with 15.2 and 18.4 percentage-point differences, respectively, after three years. In Nasarawa the discrepancy was not quite as high with an 11.2 percentage-point difference but here the overall survival estimate was more than 30 percentage points lower than at the other two sites reaching only 42% at the third survey round.

**Table 3:** **Functional survival up to three years and median survival estimates for campaign nets**

**Location**	**Survival in functional condition**			**Median survival estimate in years**
**Year**	**Crude**	**Adjusted for loss recall bias**	**Adjusted for all recall biases**	
Zamfara				
Year 1	98.9% (97.0, 99.4)	98.2% (96.7, 99.2)	98.2% (96.7, 99.2)	
Year 2	92.4% (87.6, 95.4)	90.6% (87.0, 93.5)	86.4% (82.5, 89.8)	
Year 3	89.9% (86.4, 92.5)	83.6% (79.8, 87.1)	74.7% (70.4, 78.5)	4.74 (4.40, 5.10)
Nasarawa				
Year 1	88.1% (84.3, 91.1)	84.2% (80.8, 87.4)	84.2% (80.8, 87.4)	
Year 2	69.3% (63.2, 74.8)	66.6% (61.0, 72.0)	64.8% (59.0, 69.9)	
Year 3	53.1% (46.9, 59.3)	45.0% (40.9, 50.5)	41.9% (36.7, 47.2)	2.98 (2.73, 3.21)
Cross River				
Year 1	92.0% (84.5, 96.0)	92.3% (89.5, 94.6)	92.3% (89.5, 94.6)	
Year 2	89.7% (83.8, 93.7)	87.0% (81.8, 91.4)	78.8% (74.4, 84.1)	
Year 3	88.5% (83.5, 91.9)	80.1% (75.3, 84.4)	70.1% (65.0, 74.8)	4.50 (3.93, 5.71)

Crude and recall adjusted survival estimates are shown.

Plotting the survival results against the hypothetical survival functions (Figure 2) shows that the adjusted rates follow quite closely the assumed net decay, with Zamfara consistently just below the five-year curve, Nasarawa at all time-points around the three-year curve, and Cross River around the four-year curve although with some fluctuation between the second and third data points. Calculating the median survival from two data points leads to similar results (Table 3) with a value of 4.7 years in Zamfara, 3.0 in Nasarawa and 4.5 in Cross River (based on year one and year two results).

### Use of nets

Of the campaign nets found in the survey, 60.8% had been used the previous night to sleep under and 66.5% had been used at any time in the past week. In a multivariate logistic regression model, use of the campaign net the previous night was more likely in Cross River with an adjusted odds ratio (OR) of 1.9 (p <0.0001) compared to the other sites, if the net was used over a bed frame or foam mattress compared to mat or the ground (adjusted OR 2.7, p <0.0001), if the household belonged to the highest wealth quintile (adjusted OR 1.4, p=0.005), and if there were any children under five in the household (adjusted OR 1.3, p=0.02). Use was less likely if the net had never been washed (adjusted OR 0.62, p=0.001). Interestingly, use was only marginally influenced by the physical condition of the net with a slightly increased use for nets in good condition (adjusted OR 1.2, p=0.12), but no decrease in use with increasing number of holes.

Reasons given for not using the nets were mostly based on perceptions of no threat or discomfort or dislike (84.6%), i.e., that it was too hot under the net (60.5%), that there was no malaria (28.6%), or that the net was too dirty (3.8%). Reasons that are related to condition of the net or its availability and usefulness were given for 15.4% of nets not used and the most common were that the net was too old or torn (8.5%), was currently not needed by any household member (7.3%), that the usual user of the net was not around last night (2.2%), and that net was not available due to washing (1.5%). Multiple answers were allowed for these responses.

### Washing of nets

At all three sites the proportion of nets ever washed increased over time (p <0.001) but the washing rate differed between sites, being significantly lower in Cross River with only 66.8% of campaign nets ever washed after three years compared to 93.9% in Zamfara and 90.5% in Nasarawa (p <0.0001). The wash frequency also differed between sites. Among nets that had been washed at all in the previous six months, 23.1% had been washed four or more times in Nasarawa, 12.3% in Zamfara, and only 4.8% in Cross River (p <0.0001). Slightly more than half of the campaign nets had been washed with bar soap (56.7%) while 42.2% used a detergent. Only 0.3% used bleach. There were no major differences in soap use between sites. Similarly, the pattern of drying the nets was similar with most nets (62.0%) dried on a washing line, 13.7% on the ground, 11.5% over bushes and 12.8% inside.

### Causes of damage

If a net was found to have any holes the respondents were asked whether they knew how these damages occurred. The response rate was similar in Zamfara (84.0%) and Nasarawa (85.6%) but lower in Cross River (70.2%, p=0.0001). In Zamfara and Nasarawa the response rate increased over time from 77.9% at the first survey to 87.7% at the third survey but remained constant over time in Cross River. The number of different damage mechanisms reported per net increased over time from 1.2 per damaged net in the first survey to 1.9 in the third and was consistently higher in Nasarawa with 2.2 in the three year survey compared to only 1.3 in Zamfara as well as Cross River.

**Figure 3:**
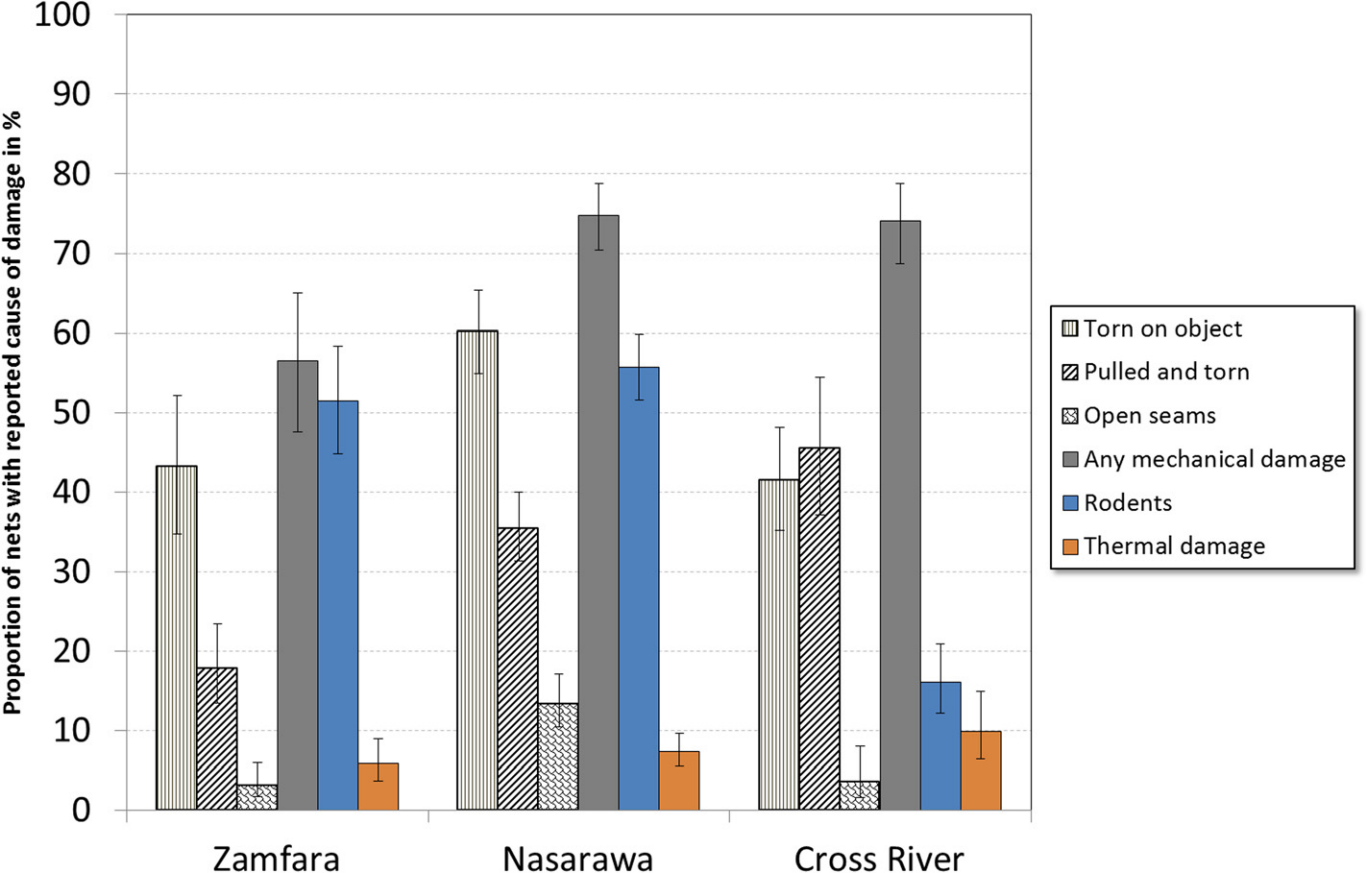
**Reported causes of damage for campaign nets with holes for which any mechanisms was reported (multiple responses possible).**

Damage patterns did not change over time but differed significantly between the three sites as shown in Figure 3. Overall mechanical damage was the dominating mechanism reported, at 56.5% in Zamfara, 74.8% in Nasarawa and 74.1% in Cross River. However, the net getting stuck on a sharp object was more often reported in Zamfara and Nasarawa, while damage by pulling on the net was more commonly reported in Cross River. Rodent damage was very high in Zamfara (51.5%) and Nasarawa (55.7%) but less common in Cross River (16.1%). Thermal damage from flames or sparks was low at all sites ranging from 5.8% in Zamfara to 9.9% in Cross River.

### Repairs

One year after the campaign the proportion of campaign nets with holes that showed any sign of repair was similarly low at all three sites (p=0.6) ranging from 6.0% in Zamfara to 10.9% in Cross River and 10.4% in Nasarawa. At year two, i.e., after a first round of BCC interventions in Nasarawa in the nested care and repair impact study, the repair rate had significantly increased in Nasarawa to 23.5% compared to 12.4% in Zamfara and 12.2% in Cross River (p=0.005) and was highest in the Nasarawa BCC intervention site (Kokona LGA) with 28.1% compared to 19.8% in the control site (Toto LGA), but this difference was not significant (p=0.14). However, after three years repair rates had also further increased in Zamfara (22.7%) and even more in Cross River (38.1%) while rates in Nasarawa remained about the same (21.0% overall, 26.5% intervention and 17.8% control). Of all the nets with any repairs, 42% had full repairs meaning that the hole in question was completely closed, 32% had both fully and partially repaired holes and 26% had only partial repairs.

**Figure 4:**
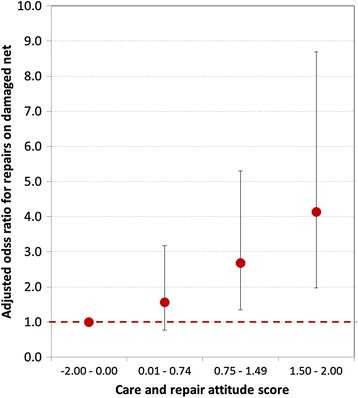
**Adjusted odds-ratio of campaign nets showing any signs of repair in relation to care and repair attitude of household respondent three years after distribution.** Adjustment variables were site and physical condition of net.

Four factors could be identified in a multivariate logistic regression analysis as independent drivers of the probability that any repairs were found on a damaged campaign net and these are shown in Table 4. Likelihood of repair increased continuously with increasing deterioration of the net and a net considered “torn” was almost three times as likely to have any repairs compared to nets in good condition. This observation was consistent across all three sites and surveys. The second factor was exposure to any messages on care and repair and this also showed a dose–response relationship, i.e., the more exposure, measured as number of care and repair messages recalled, the higher the likelihood that repairs were made. When the composite measure of the care and repair attitude score was used, which was only available for the third survey, the increase in repair behaviour with improving attitude becomes even more prominent with an adjusted OR of 4.1 (95% CI 2.0, 8.7) for households with a very positive attitude (Figure 4). Repair behaviour also improved with time since distribution, showing a doubling of the likelihood of repairs in the second year, but no further increase in the third. Finally, there was a difference in repair behaviour between the sites and, surprisingly, it was highest in Cross River although no explicit care and repair campaign had been implemented there beyond the general net-related BCC that did include some messages on “handling nets with care”. Other factors such as wealth quintile, educational level of the head of household or presence of children in the family had no impact on repair behaviour.

**Table 4:** **Determinants of repairing any holes in campaign nets with any damage across all three surveys and sites from multivariate logistic regression model (N=2,522)**

**Explanatory variable**	**Outcome: net has any repairs**		
	**OR**	**95% CI**	**p-value**
Physical condition of net			
Good (pHI 1–64)	1.00	- -	- -
Acceptable, some damage (pHI 65–300)	1.37	1.01, 1.81	0.028
Acceptable, serious damage (pHI 301–642)	1.82	1.17, 2.84	0.009
Torn (pHI 643+)	2.90	2.05, 4.10	<0.001
Exposure to information on care and repair			
None	1.00	- -	- -
1-2 times	1.32	0.92, 1.88	0.13
3-4 times	2.19	1.33, 3.62	0.002
Year since distribution			
One year	1.00	- -	- -
Two years	1.96	1.25, 3.09	0.004
Three years	2.04	1.26, 3.31	0.004
Study site			
Nasarawa control	1.00	- -	- -
Nasarawa intervention	1.40	0.96, 2.06	0.081
Cross River	2.01	1.23, 3.27	0.006
Zamfara	1.34	0.83, 2.27	0.22

pHI = proportionate hole index.

When the approximate hole surface area estimated by the pHI was compared between damaged nets with or without repairs, the median pHI was much higher for nets with repairs (657) compared to those without (199). This was driven by the fact that more damaged nets were more likely to have repairs as shown above. When the analysis was done by category of physical condition of the net, the damaged surface area was found to be exactly the same for nets with and without repairs (Table 5). A multivariate regression analysis also confirmed that this finding was consistent across surveys and sites.

**Table 5:** **Mean and median proportionate hole index by hole size category and repair status for campaign nets with any damage across all surveys and sites (N=2,522)**

**Physical condition of net**	**No repair**		**Any repair**	
	**Mean (se)**	**Median**	**Mean (se)**	**Median**
Good (pHI 1–64)	22 (0.7)	23	24 (2.1)	24
Acceptable, some damage (pHI 65–300)	168 (3.6)	178	176 (8.3)	187
Acceptable, serious damage (pHI 301–642)	490 (6.6)	491	459 (12.3)	436
Torn (pHI 643+)	2714 (95.9)	1878	2,945 (411.1)	1,694

se = standard error.

### Determinants of net integrity

A series of multivariate logistic regression models were run to explore the determinants of the physical condition of the campaign nets and how they varied between sites and over time. This showed that in general the strength of associations between physical condition and the determinants included in the models increased over time, (i.e., from the first to the third survey) as nets deteriorated and the findings were similar for the outcome of a net being in good compared to serviceable condition. Table 6 presents the result of the final model for year three that included data on the care and repair attitude score. Across the study sites, a positive attitude was associated with a higher likelihood of a net being in serviceable condition (adjusted OR 2.9, p=0.003). In Nasarawa, the site of the BCC intervention, there was also a strong dose–response relationship with increasing odds of a net being in serviceable condition as the attitude score increases (adjusted OR 2.3 for each unit of increase, p=0.002) which was absent or very weak in the other sites, so that overall the dose–response was not clearly visible (Table 6). However, the interaction term between attitude score and states was not significant (p=0.8) indicating the relationship of condition and attitude did not principally differ between states.

**Table 6:** **Determinants of surviving campaign nets being in serviceable condition three years after distribution based on multivariate logistic regression model (N=1,519)**

**Explanatory variable**	**Outcome: net in serviceable condition**		
	**OR**	**95% CI**	**p-value**
Attitude score care and repair			
Negative (−2.0-0.0)	1.00	- -	- -
Somewhat positive (0.01-0.74)	2.34	1.27, 4.32	0.007
Positive (0.75-1.49)	3.11	1.68, 5.73	<0.0001
Very positive (1.5-2.0)	2.80	1.27, 6.21	0.012
Location of net at survey day			
Hanging tied or folded	1.00	- -	- -
Hanging loose	0.63	0.43, 0.93	0.022
Taken down, not stored	0.34	0.21, 0.55	<0.0001
Stored	1.21	0.54, 2.75	0.64
Type of sleeping place			
Bed frame	1.00	- -	- -
Foam mattress on floor	0.81	0.59, 1.11	0.19
Mat	044	0.26, 0.74	0.002
Ground	0.23	0.10, 0.55	0.001
Number of children under 5 in HH			
None	1.00	- -	- -
1	0.71	0.45, 1.11	0.13
2-3	0.47	0.31, 0.70	<0.0001
4 or more	0.28	0.17, 0.45	<0.0001
Site			
Zamfara	1.00	- -	- -
Nasarawa	0.10	0.06, 0.18	<0.0001
Cross River	0.39	0.20, 0.73	0.004
Wealth quintile			
Poorest *vs* other	0.52	0.34, 0.79	0.002
Frequency of net use last week			
Every night *vs* less than every night	1.78	1.05, 3.02	0.031
Crowding			
Persons per room in HH	0.80	0.68, 0.94	0.006

Nets hanging and folded or tied up as well as those securely stored showed the best physical condition while the probability of being in serviceable condition was reduced by 37% if the net was hanging loose and by 66% if it was taken down but not stored. For the latter it was, however, not clear from the data whether this was because damaged nets were taken down and left lying around or whether they were damaged (e.g., by rodents) because they were lying around.

The presence of children aged under five in the household was associated with poorer condition of the nets and there was a statistically significant decrease in the probability of a net being serviceable with an increasing number of young children. The association with the care and repair attitude was driven by the Nasarawa data and although not different in principle (non-significant interaction term in the model) was much weaker in Zamfara and Cross River.

Nets also showed a significantly poorer physical condition if they were used over a mat (56% reduction of probability to be in serviceable condition) or on the ground (73%), if the household was in the poorest wealth quintile (48%) and if the sleeping room was crowded (20% for each additional person). Nets that were reported to have been used every day of the previous week were more likely to be in serviceable condition but here the cause and effect is most likely reversed, i.e., they were used more regularly because they were in better condition. No significant association with net integrity was found for education of head of household or the washing frequency of nets in the last six months. Finally, campaign nets from Nasarawa had a 90% lower probability to be in serviceable condition compared to those in Zamfara and – all other things being equal – were still 61% less likely to be serviceable compared to Cross River.

Determinants of reported rodent damage were assessed in a separate model which revealed four major factors: the net being used on the ground rather than a bed, mattress or mat (adjusted OR 3.6, p <0.0001); household being in the poorest wealth quintile (adjusted OR 1.7, p=0.005); food being stored in the room of the net (adjusted OR 1.6, p=0.030); and the net not hanging and not being stored (adjusted OR 1.6, p=0.047). In addition, a significant difference between sites was found with rodent damage being twice as likely in Nasarawa (adjusted OR 2.0, p=0.014) compared to Zamfara and significantly less likely in Cross River (adjusted OR 0.05, p <0.0001).

Based on the results from the models of determinants of damage, some of the factors at household and net levels that, in addition to climate and housing, were shown to impact on the net survival outcome were compared between sites and results are presented in Table 7. Somewhat surprisingly, the attitude towards care and repair three years after the campaign was similar or even slightly better in Zamfara and Cross River compared to Nasarawa even though the latter was the site for the BCC intervention and had significantly more exposure to messages on care and repair. This suggests that either households in Zamfara and Cross River had generally a better attitude towards taking care of their possessions or improvements in attitude were more easily induced by general messages on nets, or it was a combination of the two.

**Table 7:** **Differences in uni-variate analysis between sites in factors that potentially impact on net survival (survey three only)**

**Indicator**	**Zamfara**	**Nasarawa**	**Cross river**	**p-value**
Household level				
Attitude score care and repair				
Negative (−2.0-0.0)	4.2%	10.7%	6.8%	0.034
Somewhat positive (0.01-0.74)	22.8%	32.8%	31.0%	
Positive (0.75-1.49)	51.0%	45.8%	49.8%	
Very positive (1.5-2.0)	22.0%	10.7%	12.5%	
Heard any message on care and repair	21.8%	57.7%	22.0%	<0.0001
Seen rodents in house last 6 months	88.1%	95.7%	80.2%	<0.0001
Storing food in sleeping rooms	62.9%	22.0%	28.6%	<0.0001
Cooking in sleeping rooms				
Always	1.4%	0.4%	2.0%	0.047
Sometimes	15.9%	6.1%	11.4%	
Never	82.7%	93.5%	86.6%	
Mean number of under 5 per person in HH	0.30	0.20	0.17	<0.0001
Mean number of persons per room in HH	2.92	2.22	2.13	<0.0001
Net level				
Sleeping place for net				
Bed frame	59.7%	46.7%	54.9%	0.0001
Foam mattress	25.8%	38.4%	41.4%	
Mat	13.1%	9.1%	0.3%	
Ground	1.3%	5.8%	3.4%	
Position net found in				
Hanging tied or folded	43.5%	23.3%	29.0%	<0.0001
Hanging loose	34.9%	49.6%	56.2%	
Stored open	2.3%	23.6%	7.5%	
Stored in container, etc.	19.3%	3.5%	7.3%	

Generally the reported presence of rodents around the houses was very common, but least common in Cross River and highest in Nasarawa. In contrast, storing food or crops in rooms that were also used for sleeping was significantly more common in Zamfara. Cooking inside sleeping rooms was generally uncommon and differed only marginally between sites. The density of children under five as well as the crowding of people within one room was highest in Zamfara even though the mean number of persons per households was slightly lower than in Nasarawa (see Table 1).

The type of sleeping place over which the campaign nets had been used the previous night was more commonly a bed frame in Zamfara but at the same time this site had the highest proportion of nets found over mats or directly over the ground. In Zamfara, nets were also more often tied or folded up when they were hanging and securely stored when not. Nasarawa showed a low proportion of tying or folding the hanging nets and a high rate of nets not hanging and not properly stored in a box or cupboard, i.e., lying openly in the room.

## Discussion

The primary objective of this study was to apply the most recent methodology of estimating functional survival of LLINs in the field recommended by WHO [4-6] in three different areas in Nigeria using a retrospective study design in order to assess the magnitude of local variation for net survival of the same type of net (100-denier polyester LLIN). After slightly more than three years, the functional survival of the campaign nets ranged from 41.9% in Nasarawa to 70.1% in Cross River and 74.7% in Zamfara. This corresponded to an estimated median net survival of 3.0, 4.5 and 4.7 years, respectively, i.e., more than one year difference between sites.

To date only one study has been published that applies the new WHO recommendations to estimate functional net survival and this involves two brands of LLIN (one 100-denier polyester, the other 118-denier polyethylene) that were tested in one site in Cambodia [11]. After three years functional survival was 61.2% for the polyester and 58.1% for the polyethylene LLIN corresponding to a median survival of around 3.5 years.

Two other recent studies have included measures of attrition and integrity, but did not combine them into a functional survival estimate. However, both studies provide evidence of a significant variation of results between sites, similar to that observed in this study. In Benin, a 150-denier polyethylene LLIN was followed prospectively for 18 months in two sites in the north and two sites in the south of the country [9]. Attrition due to discarding and re-purposing (which is referred to as attrition due to “wear and tear” in this study), was overall 17% after 18 months with a range between sites between 10 and 32%. Even larger was the variation between sites in the proportion of surviving nets in serviceable condition, which was 52% in the poorest performing site and 82% in the best performing. Although not reported in the paper, the functional survival can be estimated from the provided information based on the same formula applied in this study as varying between 31.4 and 71.2% after 18 months, which, based on the hypothetical survival curves, would correspond to a median survival between only 1.0 and 2.2 years.

In Rwanda, LLINs were tested in three sites within the country and at each site two types of nets, one polyethylene and one polyester (no information on denier given), were sampled in neighbouring communities and prospectively followed for 24 months. Only overall attrition rates were assessed which varied between 16 and 36% after two years. While the proportion of nets in two of the sites was quite similar with around 50% still in serviceable condition for both types of nets, the third site had much poorer performance with only 10% in serviceable condition for the polyethylene LLIN and 37% for the polyester LLIN.

Two additional studies use at least a comparable methodology and both provide evidence that a median functional survival of four or more years is, indeed, possible. In rural western Kenya, a 150-denier polyethylene LLIN was studied in a cross-sectional survey five years after distribution but using actual distribution registers to verify the number of nets received [8]. The authors report an attrition rate (all causes) of 28% but this excluded households that were sampled but which no longer had any of their nets. The overall attrition after five years can, therefore, be estimated at 35 to 40%. At the same time, 61% of the surviving nets were still in serviceable condition which suggests a median survival in this environment of between 4.0 and 4.5 years. Finally, in Uganda a prospective study of a 75-denier polyester LLIN over 42 months [7] showed an all-cause attrition of 20% and nets in serviceable condition of 87% which would roughly correspond to a median functional survival of 4.5 years. In summary, these data suggest that the findings from Nigeria are plausible with respect to the between-site variation as well as the level of median functional survival of the LLIN.

The current WHO guidelines for field-testing net durability suggest prospective studies as the primary design, but also mention the possibility of retrospective studies [4]. The second objective of this study was to explore the feasibility of such a retrospective design that depends on multiple, independent, cross-sectional surveys, and to assess the magnitude of the potential recall biases that could influence the estimation of net attrition. The largest discrepancies were found between the nets reported lost and the actual loss as defined by the difference between nets received and those seen during the survey (see Table 2), although with some fluctuation over time and between sites. The bias in recall of nets received was of lesser magnitude and declined systematically with increasing time since distribution as would be expected. The overall magnitude of the recall bias, i.e., the difference between crude and adjusted functional survival estimate was very significant in two of the sites (see Figure 2 and Table 3) suggesting that without adjustment for the recall bias, results would have been very misleading. Although it cannot be said with certainty that all possible biases have been captured with the adjustments, three arguments support the view that the adjusted results are realistic. First, the adjusted survival curves as shown in Figure 2 are surprisingly well aligned with the hypothetical curves showing that at each time point the projected median survival was very similar. Second, the major decline and differences between sites in functional survival were driven by the net integrity, which was directly observed and not subject to recall bias. Third, the estimated all-cause attrition rates after adjustment (see Table 2) agree quite well with those reported in the literature. Batt *et al.* [22] report a 21% attrition rate from India after three years based on a prospective follow-up. Similarly, Fettene *et al*. [23] report a rate of 28% after two to three years in Ethiopia, and from a study by Hassan *et al.* [24] in Sudan, attrition of 19% after 18 months can be estimated. The previously mentioned prospective study from Rwanda [10] found between 16 and 36% after 24 months and the one from Kenya [8] around 40% after five years. Other studies report slightly higher rates of 20% attrition after 12 months in Uganda [25] and Liberia [26], 43% after 18 months in Benin [9] and 45% after 24 months in Sudan [27] while another publication reports a low rate of only 20% after 42 months from a prospective study in Uganda [7]. However, while the results from the recall-adjusted retrospective estimates of durability from this study seem feasible, some uncertainty remains and a prospective approach would always be preferable.

The major cause of holes reported by the survey respondents was mechanical damage at all three sites affecting 57 to 75% of all damaged nets, followed by holes caused by rodents, which were frequent in two sites (Zamfara 51% and Nasarawa 56%), but much lower in the third (Cross River 18%). The third damage category, thermal damage from burns or sparks, was generally low, less than 10%, and similar at all sites. This order of magnitude of the three major mechanisms of damage, primarily mechanical failures followed by animal and thermal damage, have been confirmed in the only laboratory-based textile analysis of over 500 damaged nets randomly sampled from seven sites in Africa and Asia recently presented to a WHO consultation on net durability (Russell, pers comm). A sub-sample of year two nets from all three sites of this study was part of that textile analysis of damage and the Nigeria data from the laboratory mostly confirm the pattern between sites and the order of damage causes expressed as hole frequency by mechanism: mechanical damage was 45% of all holes in Zamfara, 79% in Nasarawa and 66% in Cross River, while rodents were most common in Zamfara 51%, but low in Cross River (25%) and Nasarawa (17%). Thermal damage was only between 3 and 5% of all holes (Wheldrake, pers comm). This suggests that determination of damage by interview of net owners is not precise, but gives a reasonably exact idea of the dominating mechanisms of damage in an area.

Three other studies have attempted to capture the causes of damage quantitatively from household interviews and found similar results. The previously mentioned study from Benin [9] reports 84% mechanical damage after 18 months, 11% thermal damage (but with significant variation between sites of 2 to 29%) and 2% from rodents. In a cross-sectional sample of nets from various sources and ages Mutuku and colleagues report 63% mechanical damage (excluding “don’t know” responses), 12% from animals and 12% from “fire” at the Kenyan coast near Mombasa [12]; and from a refugee camp in Western Uganda, Spencer *et al*. [25] report after 12 months, 46% of damage to be from rats, 24% from tears and 8% from burns. A more semi-quantitative assessment is reported by Picado *et al*. [28] who state that in India most damage was reportedly caused by animals (dogs, goats and rats), while in Nepal the most common cause was mechanical damage from nails and sticks. Other publications only mention the causes of damage reported by respondents without quantifying, but generally agree that mechanical, rodents and thermal are the major causes [21,27,29-33].

The analysis of determinants of damage in this study revealed a number of factors that are associated with poverty (poor housing, crowding, absence of adequate sleeping places, poorest wealth quintile) as well as behavioural aspects such as letting the net hang loose during the day, not storing it properly when not in use (rodents), having food or crops stored in the same room (rodents), having young children with access to the bedroom, and the general attitude of the household towards net care and repair. Particularly for the Nasarawa site, these mechanisms were confirmed by qualitative research where members in focus group discussions mentioned “children, rodent, everyday handling that is not gentle and characteristic of the sleeping place” as the main causes of damage [16]. Similar findings also are reported from Senegal [15] and the association of poor housing [34] and storage of food [35] with increased rodent presence is also well documented. While the aspects of care and repair attitudes has been discussed in more detail in the context of the BCC impact study results [18], it is noteworthy here that attitude scores increased at all three sites even though no explicit BCC activities had been undertaken in Zamfara and Cross River. However, some level of exposure to net-related BCC messages is likely to have occurred in these states also, as they are part of the USAID funded Malaria Action Plan for States (MAPS). This, in combination with a higher level of net culture that had previously been described for the north and southeast [36], might well explain the observed improved care and repair attitude over time. Overall, the differences in living conditions and attitudes (see Table 7) between the sites explains the significant variation in estimated net survival between the sites, which would most likely have been even bigger had there not been an intensive BCC campaign in Nasarawa.

In the past, the discussion on improvement of net care and repair has very much focused on calls for repair of nets [37,38] based on generally low levels of observed repairs made of less than 20% of damaged nets [12,22,37-40], although in some cases rates between 30 and 64% have been found [41-44]. In addition, there is some evidence that repair behaviour can be induced by BCC [45]. This latter observation can be confirmed by the present study where rates of repair increased from around 10% one year after net distribution to between 21 and 38% after three years, being driven by care and repair attitude and the level of damage on the net. However, this study, for the first time, also looked at the impact of repairs on the holed surface area and found no detectable difference. Although the use of the pHI can only be considered a rough approximation of damaged area due to its underlying assumptions and potential measurement errors, this does suggest that a strategy that focuses more on prevention of holes rather than attempts to fix damage may be more promising.

## Conclusions

Differences of more than one year in estimated median survival of a 100-denier polyester LLIN between three areas of Nigeria were driven by living conditions and household behaviour and attitudes, providing evidence that where and how an LLIN is used is at least as important for durability as the textile design or structure of the net.

Recall bias in a retrospective durability study can be significant and while adjustments can be made, some uncertainty remains such that prospective studies on durability are preferable wherever possible.

Repair of damaged nets can be induced by improved attitude towards care and repair, but does not seem to measurably improve net condition. Focus should, therefore, be on preventive behaviour that protects the net from damage, such as folding or tying the net up every day, keeping children away, avoiding storing food or crops in the same room, and storing the net safely when not in use.

## Additional files

Additional file 1:
**Households sampled and nets assessed at different locations and time points.** Targeted and achieved sample for households and nets at each site and time point. Additional file 2:
**Flow diagram of adjustment process for recall bias of nets received and lost.** A step-by-step presentation of calculations of adjustment for recall biases. Additional file 3:
**Hypothetical loss functions with defined median survival.** Annually remaining nets in the hypothetical loss functions that show the median survival and can be used to re-create the graphs. Additional file 4:
**House characteristics and socio-economic background by site.** Details of household background characteristics comparing sites and using pooled data from all three surveys.
