# Supplementary material for: Field durability of the same type of long-lasting insecticidal net varies between regions in Nigeria due to differences in household behaviour and living conditions
Source: Malar J. 2015 Mar 24;14:123. doi: 10.1186/s12936-015-0640-4 (PMC4376338; doi:10.1186/s12936-015-0640-4)
Supplement: Additional file 1: — Households sampled and nets assessed at different locations and time points. Targeted and achieved sample for households and nets at each site and time point. [file 12936_2015_640_MOESM1_ESM.pdf]

Additional file 3

Table 9: Households sampled and nets assessed at different locations and time points

Location and Year	Years since campaign	Households		Campaign nets	
		Target	Sampled	Found	Assessed for holes
Zamfara (Shinkafi LGA)					
Year 1	1.1	300	300 (100%)	734	715 (97.4%)
Year 2	2.1	300	291 (97.0%)	493	489 (99.2%)
Year 3	3.1	300	298 (99.3%)	461	435 (94.4%)
Zamfara Total	n.a.	900	889 (98.8%)	1688	1639 (97.1%)
Nasarawa (Toto LGA)					
Year 1	1.2	300	278 (92.7%)	457	425 (93.0%)
Year 2	2.3	300	299 (99.7%)	482	463 (96.1%)
Year 3	3.3	420	412 (98.1%)	560	522 (93.2%)
Sub-Total	n.a.	1020	989 (97.0%)	1499	1410 (94.1%)
Nasarawa (Kokona LGA*)					
Year 1	1.2	300	276 (92.0%)	397	376 (94.7%)
Year 2	2.3	300	297 (99.0%)	436	410 (94.0%)
Year 3	3.3	300	297 (99.0%)	358	327 (91.3%)
Sub-Total	n.a.	900	870 (96.7%)	1191	1113 (93.5%)
Nasarawa Total	n.a.	1920	1859 (96.8%)	2690	2523 (93.8%)
Cross River (Abi LGA)					
Year 1	1.1	300	301 (100%)	482	398 (82.6%)
Year 2	2.2	300	300 (100%)	403	376 (93.3%)
Year 3	3.1	300	300 (100%)	406	380 (93.6%)
Cross River Total	n.a.	900	901 (100%)	1291	1154 (89.4%)
Overall					
Year 1	n.a.	1200	1155 (96.3%)	2070	1914 (92.5%)
Year 2	n.a.	1200	1187 (98.9%)	1814	1738 (95.8%)
Year 3	n.a.	1320	1307 (99.0%)	1785	1664 (93.2%)
Overall Total	n.a.	3720	3649 (98.1%)	5669	5316 (93.8%)

* Intervention site for care and repair study
