# Supplementary figures and images for: Field durability of the same type of long-lasting insecticidal net varies between regions in Nigeria due to differences in household behaviour and living conditions
Source: Malar J. 2015 Mar 24;14:123. doi: 10.1186/s12936-015-0640-4 (PMC4376338; doi:10.1186/s12936-015-0640-4)

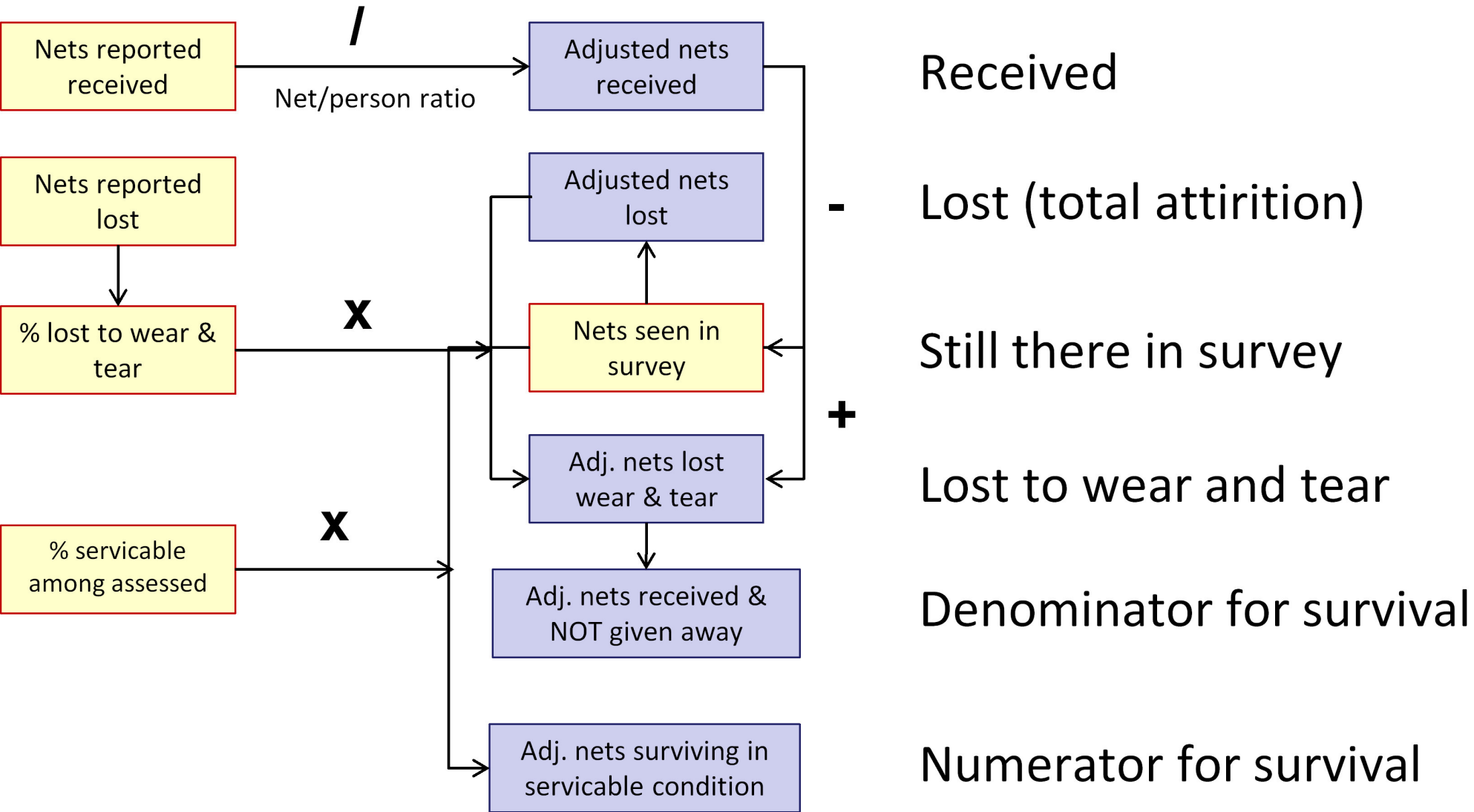

Calculate 95% CI from exact binomial inflated by design effect

Supplement: Additional file 2: — Flow diagram of adjustment process for recall bias of nets received and lost. A step-by-step presentation of calculations of adjustment for recall biases. [file 12936_2015_640_MOESM2_ESM.pdf]
