# Supplementary material for: Field durability of the same type of long-lasting insecticidal net varies between regions in Nigeria due to differences in household behaviour and living conditions
Source: Malar J. 2015 Mar 24;14:123. doi: 10.1186/s12936-015-0640-4 (PMC4376338; doi:10.1186/s12936-015-0640-4)
Supplement: Additional file 3: — Hypothetical loss functions with defined median survival. Annually remaining nets in the hypothetical loss functions that show the median survival and can be used to re-create the graphs. [file 12936_2015_640_MOESM3_ESM.pdf]

Additional file 2

Table 8: Hypothetical loss functions with defined median survival. Shown are the percentages of originally distributed nets still present

**Median survival**	**Years since distribution**																		
	**0**	**1**	**2**	**3**	**4**	**5**	**6**	**7**	**8**	**9**	**10**	**11**	**12**	**13**	**14**	**15**	**16**	**17**	**18**
1-year	100	50	4	0															
1.5-year	100	74	28	4	0														
2-year	100	85	50	19	4	0													
2.5-year	100	90	65	36	15	4	1	0											
3-year	100	93	74	50	28	12	4	1	0										
3.5-year	100	95	80	60	40	23	11	4	1	0									
4-year	100	96	84	68	50	33	19	10	4	2	0								
4.5-year	100	97	87	74	58	42	28	17	9	4	2	1	0						
5-year	100	97	90	78	64	50	36	24	15	8	4	2	1	0					
5.5-year	100	98	91	82	69	56	43	31	21	14	8	4	2	1	0				
6-year	100	98	93	85	74	62	50	39	29	20	13	8	5	3	1	1	0		
6.5-year	100	98	94	87	78	67	56	45	35	26	19	13	8	5	3	2	1	0	
7-year	100	99	95	88	80	71	61	50	41	32	24	17	12	8	5	3	2	1	0
