# Supplementary material for: Field durability of the same type of long-lasting insecticidal net varies between regions in Nigeria due to differences in household behaviour and living conditions
Source: Malar J. 2015 Mar 24;14:123. doi: 10.1186/s12936-015-0640-4 (PMC4376338; doi:10.1186/s12936-015-0640-4)
Supplement: Additional file 4: — House characteristics and socio-economic background by site. Details of household background characteristics comparing sites and using pooled data from all three surveys. [file 12936_2015_640_MOESM4_ESM.pdf]

Additional file 4

Table 10: House characteristics and socio-economic background by site (pooled data across all three surveys); Est.= estimate; HH=household; U5=child under five years

**Indicator**	**Zamfara** (N=896)		**Nasarawa** (N=1896)		**Cross River** (N=902)	
	Est.	95% CI	Est.	95% CI	Est.	95% CI
House characteristics						
% of houses with sheet roof	32.4%	20.9 , 46.4	89.8%	82.9 , 94.2	85.1%	80.7 , 88.7
% of houses with brick/plaster walls	15.6%	8.2 , 27.6	42.9%	37.3 , 48.6	75.6%	69.0 , 81.2
% of houses with earth/clay floors	74.4%	62.0 , 83.9	42.3%	35.4 , 49.5	24.0%	18.9 , 29.9
% of houses using firewood to cook	98.9%	95.6 , 99.7	99.0%	98.0 , 99.4	84.4%	77.9 , 89.2
% of HH with access to safe water	78.8%	69.8 , 85.6	24.2%	17.3 , 32.7	60.0%	48.2 , 70.7
% of HH with any latrine	85.4%	76.3 , 91.4	34.3%	28.0 , 41.2	72.0%	65.4 , 77.7
Household assets						
% of HH owning a radio	81.9%	76.9 , 86.1	82.5%	79.0 , 85.5	74.2%	69.7 , 78.2
% of HH owning a mobile phone	63.6%	57.1 , 69.7	72.1%	67.9 , 76.0	76.6%	72.3 , 80.4
% of HH owning a TV	14.1%	7.1 , 25.9	28.8%	24.6 , 33.4	65.2%	58.9 , 71.0
% of HH owning means of transport	63.2%	56.9 , 69.0	84.4%	81.0 , 87.4	53.9%	49.5 , 58.3
% of HH owning land	95.0%	87.1 , 98.2	97.1%	95.0 , 98.4	89.0%	85.3 , 91.9
% of HH owning any husbandry	82.1%	72.1 , 89.1	90.8%	88.8 , 92.5	45.8%	42.0 , 81.9
Household demographics						
% of HH polygamous	75.9%	68.6 , 81.9	46.6%	42.9 , 50.4	43.7%	37.7 , 49.9
% of HH with any U5	81.9%	78.4 , 85.0	75.0%	71.9 , 77.8	60.0%	55.6 , 64.2
Education of head of HH						
Non-literate	55.6%	47.0 , 63.8	52.4%	47.5 , 57.2	23.6%	20.2 , 27.4
Primary	27.7%	20.8 , 35.8	20.6%	18.1 , 23.4	31.0%	26.8 , 35.6
Secondary or better	16.7%	11.2 , 24.7	27.0%	23.5 , 30.9	45.3%	40.1 , 50.7
